# Mammary Epithelial Cell Spheroid: Stabilization Through Vascular-Wall Mesenchymal Stem Cells and Endothelial Cells Co-Culture

**DOI:** 10.3390/ani15213095

**Published:** 2025-10-24

**Authors:** Debora La Mantia, Roberta Salaroli, Biljana Petrovic, Domenico Ventrella, Augusta Zannoni, Monica Forni, Chiara Bernardini

**Affiliations:** 1 Department of Veterinary Medical Sciences, University of Bologna, 40064 Bologna, Italy; roberta.salaroli@unibo.it (R.S.); domenico.ventrella2@unibo.it (D.V.); augusta.zannoni@unibo.it (A.Z.); chiara.bernardini5@unibo.it (C.B.); 2 Centre for Applied Biomedical Research (CRBA), University of Bologna, 40126 Bologna, Italy; biljana.petrovic2@unibo.it; 3 Health Sciences and Technologies-Interdepartmental Center for Industrial Research (CIRI-SDV), Alma Mater Studiorum—University of Bologna, 40126 Bologna, Italy; monica.forni@unibo.it; 4 Department of Medical and Surgical Sciences, University of Bologna, 40138 Bologna, Italy

**Keywords:** mammary gland spheroids, Göttingen Minipigs, endothelial cells, vascular-wall mesenchymal stem cells, triple 3D model, complementary methods

## Abstract

Over the past decade, the interest in using 3D cell culture models for studying the mammary gland in biomedical and veterinary fields has increased, but a fully functional in vitro model for domestic species is still lacking. Multiple cellular components, including epithelial cells, vascular endothelial cells, and stromal/stem cells, sustain the secretory mammary gland tissue in a well-organized 3D architecture. Considering the Göttingen Minipigs widely used for translational lactation studies, this work aimed to establish a 3D culture protocol to generate mammary heterogeneous multicellular spheroids composed of three different Göttingen Minipigs primary cells: mammary epithelial cells (mpMECs), aortic endothelial cells (mpAECs), and vascular-wall mesenchymal stem cells (mpVW-MSCs). Cells were cultured with hanging-drop (HD) and ultra-low-adherence plate (ULA) methods, evaluating aggregate formation in both monocultures and co/triple co-cultures. Brightfield area, eccentricity, viability, and cell distribution were analyzed. Results showed mpMECs formed irregular aggregates in both HD and ULA, while more compact and viable spheroids were formed when co-cultured with mpVW-MSCs and mpAECs by ULA. A well-organized cellular distribution was demonstrated by cytokeratin-18, vimentin, and e-NOS immunofluorescence analysis. In conclusion, this study established a stable 3D mammary multicellular spheroid model, representing a promising tool for future studies on hormonal modulation and mammary gland physiology.

## Simple Summary

It has been widely demonstrated that vascular-wall multipotent cells are particularly involved in vascular-remodeling processes, especially when interacting with endothelial cells, by contributing to blood vessel stabilization and maturation. In the mammary alveolus, the vascular wall cells, including endothelial cells, are crucial for maintaining the blood–milk barrier by supporting the function of mammary epithelial cells. Considering that, to date, there is a shortage of 3D co-culture models that mimic in vivo mammary glandular structure for domestic species, in the present research, we described a 3D co-culture model where primary mammary epithelial cells from Göttingen Minipigs were co-cultured with the two vascular-wall cellular populations: the mesenchymal stem cells and endothelial cells. The results showed that co-culturing the mammary epithelial cells with the two vascular wall cell populations led to the formation of more stable mammary gland multicellular spheroids with a well-organized cell structure, offering a strong potential for various applications in veterinary research, including lactation biology and regenerative approaches.

## 1. Introduction

Mesenchymal stem/stromal cells (MSCs) have garnered significant attention in regenerative medicine applications and tissue engineering, thanks to their multipotency and ability to support tissue repair. MSCs reside in various tissues and are known to contribute to homeostasis, immunomodulation, and wound healing [1,2,3]. Moreover, the mesenchymal stromal cells play a crucial role in maintaining the microenvironment necessary for stem cell function and differentiation by providing structural support, secreting bioactive molecules, and mediating cell–cell interactions that influence the fate of MSC [4].

Different types of stem cells reside in adult mammary glands, including MSCs, which are principally involved in the vascular reservoir. In particular, MSCs secrete a broad range of pro-angiogenic factors, such as VEGF and FGF, promoting endothelial cell proliferation and vessel stabilization. Additionally, MSCs interact with perivascular niches, supporting vascular integrity and facilitating neovascularization during mammary gland development and lactation. Their immunomodulatory properties also help maintain a microenvironment conducive to vascular homeostasis. Thus, MSCs are essential cellular components orchestrating vascular support in mammary tissue dynamics [5,6]. Among the different populations of MSCs, the vascular-wall mesenchymal stem cells (VW-MSCs) are predominantly found in perivascular niches of blood vessels across nearly all tissues, where they exhibit a pericyte function and are particularly involved in vascular remodeling processes, in a dynamic system, by contributing to the vascular homeostasis [3,5,7,8,9,10]. VW-MSCs were isolated from large vessels of different species, including humans [11], rodents [12,13], and porcine species [14,15]. It has also been shown that vascular-wall multipotent cells can promote blood vessel stabilization and maturation, particularly during interactions with endothelial cells (ECs) within the vascular network [16,17]. In particular, during the period of lactation, angiogenesis plays a key role in alveolar development and facilitates optimal milk production. A continuous cross-talk involving the VEGF pathway between endothelial cells and VW-MSCs is crucial for correct vascular remodeling [6,18]. Porcine Vascular Wall Mesenchymal Stem Cells (pVW-MSCs) showed an extraordinary ability to sustain a capillary-like tube formation through a physical interaction with EC, but also by secretion of pro-angiogenic factors [15,19]. Moreover, VW-MSCs can directly influence MECs via paracrine signaling by factor secretion or extracellular vesicles (EVs), such as exosomes, and by a direct cell–cell contact through gap junctions or adhesion molecules [20,21].

The vascular network of the mammary gland plays a critical role in ensuring adequate perfusion and metabolic support, particularly at the alveolar level. Alveoli, the functional units responsible for milk synthesis and secretion, require a rich capillary supply to sustain high rates of nutrient and oxygen exchange. This vascularization facilitates the delivery of essential substrates, such as glucose, amino acids, and lipids, necessary for lactogenesis. Moreover, the close interplay between the capillary endothelium and alveolar epithelium supports efficient hormonal signaling, particularly involving prolactin and oxytocin. Thus, the integrity and adaptability of the mammary vasculature are fundamental to both the development and sustained function of lactating tissue [22,23]. A complete and correct angiogenesis is crucial for lactation and maintaining the integrity of the blood–milk barrier is, therefore, essential for the health of both the mammary gland and the offspring that rely on the milk [23,24]. Over the past decade, interest in 3D cell culture models of the mammary gland has increased, both for biomedical applications, given the high incidence of human breast cancer, and for veterinary research on milk production, disease mechanisms, and animal health. To date, a fully defined in vitro model that better mimics in vivo mammary gland is lacking [25]. Various 3D cell culture systems based on mammary epithelial cell lines were tested, ranging from the use of Matrigel and ultra-low attachment culture supports to hanging-drop culture, and were then compared with traditional 2D cell culture [26,27]. Our research group previously developed a mammary epithelial cell culture method to investigate epithelial barrier integrity. This was achieved by culturing MECs on specific porous membrane supports, a strategy successfully employed with both pig/minipig [28,29] and human primary cells [30]. The need to develop advanced in vitro models for the porcine mammary gland arose within the European project IMI ConcePTION (https://www.imi-conception.eu/, accessed on 21 October 2025), for which our research group has already contributed to the development of an in vivo model and an in vitro model under traditional 2D culture conditions. To recreate a 3D multicellular structure with features similar to the mammary alveolus, this study aimed to develop a triple 3D culture model, comparing two different methods, in terms of time, spheroid formation, number, area, and viability of spheroids. This model involved co-culturing heterogeneous populations of minipig mammary epithelial cells (mpMECs) with minipig VW-MSCs (mpVW-MSCs) and aortic endothelial cells (mpAECs).

## 2. Materials and Methods

### 2.1. Chemicals and Reagents

Dulbecco phosphate-buffered saline (DPBS) with calcium and magnesium, Fetal Bovine Serum (FBS), trypsin-EDTA, antibiotic–antimycotic 100× (cod. 15240062), recombinant human epidermal growth factor (hEGF), Dulbecco’s Modified Eagle Medium: Nutrient Mixture F-12 (DMEM/F12), human endothelial serum-free medium (hESFM, cod. 11111044), and alamarBlueTM cell viability reagent (cod. DAL1025) were purchased from Thermo Fisher Scientific (Waltham, MA, USA). Dimethyl sulfoxide (DMSO) and Fluoroshield^TM^ with DAPI histology mounting medium were purchased from Sigma-Aldrich (St. Louis, MO, USA). All plastic supports for adherent cell culture and the 96-well round-bottom ultra-low attachment plates (cod. 7007) were purchased from Corning-Beckton-Dickinson (Franklin Lakes, NJ, USA). Pericyte Growth Medium (PGM) and the specific supplement mix were purchased from PromoCell (PromoCell GmbH, Heidelberg, Germany). The medium used for adherent cell culture of mpMECs, named the Expansion Medium (Em), was composed of DMEM/F12 with 10% FBS, 5 μg/mL insulin and 0.5 μg/mL hydrocortisone (both provided by Promo Cell, Heidelberg, Germany), 5 ng/mL hEGF (provided by Thermo Fisher), and 1% anti-anti (provided by Thermo Fisher). The medium used for mpAECs cultures, named endothelial cell medium (EC medium), was composed of human endothelial serum-free medium with 5% FBS and 1% antibiotic-antimycotic solution. The medium used for mpVW-MSCs cultures, named Pericyte growth medium (PGM), was composed of a specific pericyte growth medium with supplement mix and 1% antibiotic–antimycotic solution. The medium used for spheroid 3D cultures was Expansion Medium without FBS, named serum-free Em. The antibodies used for the immunofluorescence analysis are listed in Table 1.

### 2.2. Culture of mpMECs, mpAECs, and mpVW-MSCs

Primary cell cultures of minipig mammary epithelial cells (mpMECs) were isolated and characterized as previously described in detail [29]. mpMECs were obtained from three sows that showed resting mammary glands and derived via mammary interlobular and intralobular ducts [29]. mpMECs, at passages 2–5, were seeded and cultured in T-25 primary culture flasks (2 × 10^4^ cells/cm^2^) in the Em medium. Primary cultures of minipig aortic endothelial cells (mpAECs) and minipig vascular wall–mesenchymal stem cells (mpVW-MSCs) were isolated from the thoracic aorta, characterized as previously described [31], and used at the third passage. mpAECs were seeded and cultured in T-25 primary culture flasks (2 × 10^4^ cells/cm^2^) in EC medium. mpVW-MSCs were seeded and cultured in the T-25 primary culture flask (2 × 10^4^ cells/cm^2^) in PGM. The cells were maintained at 5% CO_2_ and 38.5 °C until 70% confluence was achieved before starting the 3D culture. An inverted Nikon Eclipse Microscope (TS100) (Tokyo, Japan) with a digital C-Mount Nikon photo camera (TP3100, Tokyo, Japan) was used to monitor the cell morphology.

### 2.3. Three-Dimensional Culture by Hanging-Drop Assay

To set up a 3D cell culture protocol and to create a mammary multicellular spheroid, the cells were detached from flasks, collected, counted, and the hanging-drop assay was applied. mpMECs were seeded as a monoculture or by combining mpMECs with mpVW-MSCs and mpAECs in serum-free Em. The cells (mpVW-MSCs-mpAECs) were mixed as follows: A 1:10 ratio was previously prepared [15]. Then, a 1:10 ratio of mpMECs (mpVW-MSCs–mpAECs) was mixed. Drops of 10 μL at 1 or 2 × 10^4^ cells/drop were seeded on the cover of a Petri tissue culture dish of 60 mm (6 drops/Petri dish). DPBS (10 mL) was placed at the bottom of the dish to create a hydration chamber and prevent the drops from drying out. Then, the cover was overturned and incubated at 5% CO_2_, 38.5 °C, and a humid environment. After 24 h, the resulting cellular aggregates were observed by means of an inverted Eclipse Microscope (TS100) with a digital C-Mount Nikon photo camera (TP3100). Analysis of the Brightfield area of spheroids was performed with ImageJ Fiji software (version 2.16.0).

### 2.4. Three-Dimensional Culture in Low-Adherent Plate and Long-Term Culture

The formation of spheroids was also tested by seeding mpMECs in monoculture or by combining mpMECs with mpAECs and mpVW-MSCs using the 96-well round-bottom ultra-low-attachment plates. Briefly, 100 μL of serum-free Em containing a total of 5 × 10^3^ or 10 × 10^3^ cells/well were seeded in each well at the following cell type combinations and proportions: monoculture of mpMECs, co-culture of mpMECs–mpVW-MSCs (2:1), and triple co-culture of mpMECs–mpVW-MSCs–mpAECs (3:1:2). The cell-mixing ratio for the spheroids culture model was taken by Paris and colleagues [32]. Immediately after seeding, the plates were centrifuged at 300× *g* for 5 min and incubated in the Incucyte^®^ S3 live-cell imaging system (Sartorius, Göttingen, Germany) with S3/SX1 G/R Optical Module. The instrument was housed inside the 5% CO_2_ incubator at 37 °C and in a humid environment. Incucyte technology offers real-time live-cell imaging and analysis for continuous monitoring of cell behavior inside the incubator, by providing a wide range of quantitative data on the objects examined. Brightfield images were acquired using 4× magnification every 3 h from seeding to 7 days. The Brightfield object area expressed in µm^2^ was determined from images analyzed. The eccentricity of the object analysis was also examined and compared with the Brightfield object average eccentricity parameter calculated by the Incucyte^®^ Spheroid Analysis Software (Incucyte 2023ARev2). The metric is expressed in a range from 0 to 1, where objects having a value of 0 represent a perfect circle. Volume reconstruction of cell masses was obtained by using ImageJ Fiji software and ReVisp software (ReViSP v2.3) [33].

### 2.5. Viability Assay

To determine the viability of spheroids, the AlamarBlue assay was performed. This is a resazurin-based technique that quantifies the metabolic activity of viable cells. Briefly, the assay is based on the principle that resazurin (blue, non-fluorescent dye) is reduced to resorufin (pink, highly fluorescent dye), due to intracellular redox status, in metabolically active cells. Following 7 days of 3D culture in low-adherent plates, the spheroids were transferred to black with clear flat-bottom 96-well plates. AlamarBlue^TM^ reagent was added, as indicated by manufacturer instructions, and incubated for at least 18 h at 37 °C, 5% CO_2_. The fluorescence of resorufin was detected by 560 nm excitation and 590 nm emission, with an excitation bandwidth of 12 nm, using the Varioskan^TM^ LUX multimode microplate reader (ThermoFisher Scientific).

### 2.6. Immunofluorescence Analysis

To analyze the distribution of three different cell types on multicellular spheroids, cells cultured on low-adherent round-bottom plates were fixed to perform the immunofluorescence analysis. After washing with DPBS, spheroids were fixed in 4% paraformaldehyde for 45 min at room temperature (RT). Blocking of unspecific epitopes followed fixation with 10% FBS in DPBS for 1 h at RT. Two washes in DPBS of 10 min were performed, and then the cells were incubated with the appropriate primary antibody (Table 1) diluted in 0.3% Triton-X 100 and 2% FBS in DPBS, for 48 h at 4 °C. After being rinsed in DPBS (3 times, 15 min each), the cells were incubated with fluorochrome-labeled secondary antisera diluted in DPBS for 1 h at RT (Table 1). After 3 washes (15 min each), spheroids were transferred to slides, and a mounting medium with DAPI was used to counterstain the nuclei. Stained cells were observed using a confocal laser scanning microscope (Nikon A1R, Amstelveen, The Netherlands). Analysis of images was performed with NIS Element AR software (version 5.20.01).

### 2.7. Statistical Analysis

A Shapiro–Wilk test was applied to test for normal distribution. To analyze the data of aggregates’ area obtained using the hanging-drop method, a Wilcoxon matched-pairs signed-rank test was applied. To analyze area and eccentricity data generated by the ULA 3D culture method, a Kruskal–Wallis test, with a Dunn’s multiple comparison test (*p* < 0.05), was applied. Data obtained via the viability assay were analyzed using a one-way ANOVA with Tukey’s multiple comparison test between groups (*p* < 0.05) (GraphPad Prism 9, version 9.1.0). All data analyzed are representative of at least three independent experiments, each consisting of three or eight technical replicates.

## 3. Results

### 3.1. Cell Morphology and Formation of Cellular 3D Aggregates via Hanging-Drop Method

**Figure 1:**
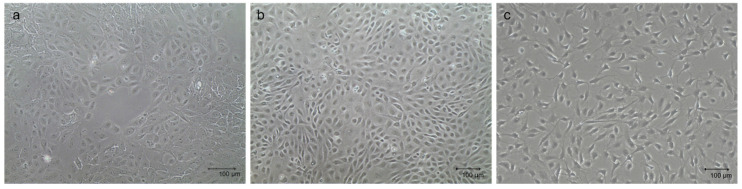
Representative images of mpMECs (**a**), mpAECs (**b**), and mpVW-MSCs (**c**). Morphology when ~70% confluence is reached. Scale bar 100 µm.

The three primary cellular populations, which were thawed and maintained in monolayer before setting up the multicellular 3D culture model, displayed their typical tissue-derived morphology, as previously described [29,31]. Specifically, mpMECs showed an epithelial cobblestone morphology (Figure 1a), mpAECs exhibited the polygonal endothelial morphology (Figure 1b), and mpVW-MSCs exhibited the elongated spindle-shaped fibroblast-like morphology, typical of a perivascular mesenchymal cell morphology described when cultured in PGM (Figure 1c) [31].

**Figure 2:**
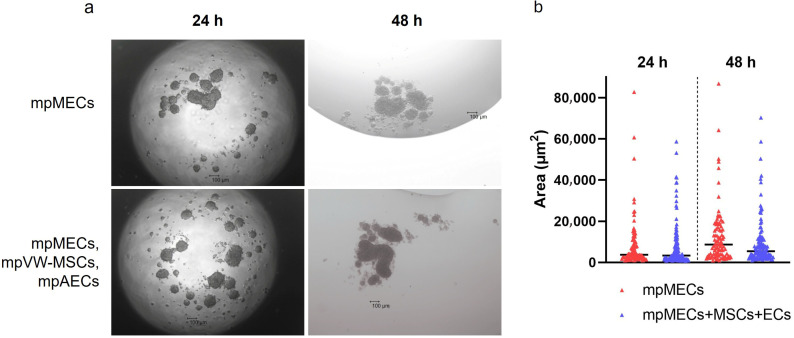
The formation of mammary epithelial cell aggregates when cultured via hanging drop as monoculture (mpMECs) or triple co-culture with vascular-wall mesenchymal stem cell (mpVW-MSCs) and endothelial cells (mpAECs). (**a**) Images are representative morphology of 3D cell aggregates at 24 h and 48 h performed at 4× objective (scale bar 100 µm). (**b**) Area of the cellular aggregates represented by a scatter dot plot graph, where the central segment was the median.

When cultured in 3D and serum-free culture conditions utilizing the hanging-drop method, mpMECs aggregated within 24 h, forming three-dimensional structures irregular in shape of various sizes. No difference in the area of objects analyzed was evidenced at two seeding densities (1 or 2 × 10^4^ cells/drop). To analyze the influence of the vascular wall mesenchymal stem cells and endothelial cells on mpMECs, mpMECs were co-cultured with the two vascular cell populations: mpAECs and mpVW-MSCs. No difference in 3D structures formed and in the area of objects analyzed between the monoculture of mpMECs and triple co-culture was shown (Figure 2a,b). The cells continued to aggregate until degeneration after 48 h (Figure 2a).

### 3.2. Culture in Low Adherence Round-Bottom Plates and Spheroid Formation

**Figure 3:**
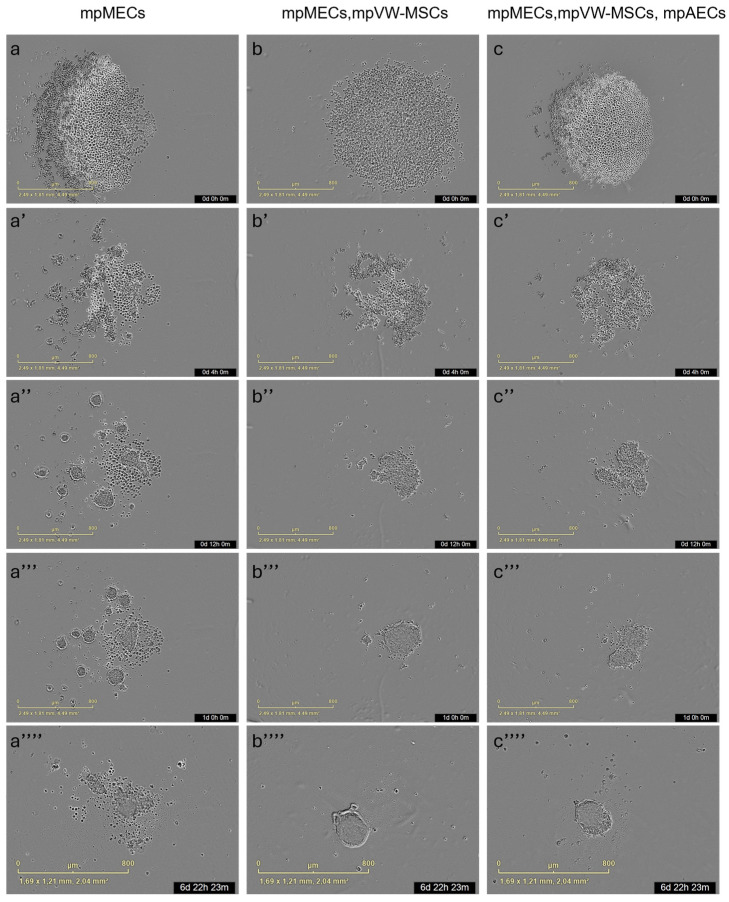
Representative images of 3D culture of the three groups: mpMECs (**a**–**a’’’’**), co-culture mpMECs-mpVW-MSCs (**b**–**b’’’’**), and triple co-culture mpMECs-mpAECs-mpVW-MSCs (**c**–**c’’’’**). Individual panels are the scans carried out at the different time points performed at Incucyte live-imaging technology.

**Figure 4:**
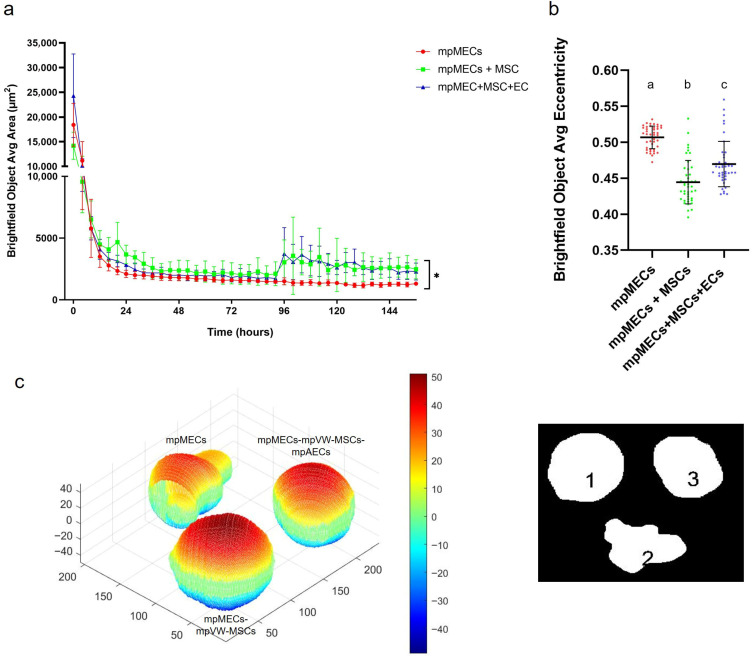
(**a**) Area of Brightfield objects analyzed at Incucyte S3 live-imaging system, from Time 0 (T0—at seeding) until 7 days of culture in low-attachment round-bottom plates; (*) indicates significant differences at *p* < 0.05. (**b**) Eccentricity parameter (range 0–1) calculated at the day 7 of culture in the three different cultures is represented as scatter dot plot, where the horizontal lines are the mean ± SD of eight technical replicates of at least three independent experiments (circles), and letters above represent the differences between groups (*p* < 0.05) when the Kruskal–Wallis test with Dunn’s multiple comparison test was applied. (**c**) Volume reconstruction of the spheroids after 7 days of culture, on the left, obtained by the mask of area determined by ImageJ Fiji on the right, assessed by ReViSP software (scale in voxels) (1 refers to mpMECs, 2 refers to mpMECs-mpVW-MSCs and 3 refers to mpMECs-mpVW-MSCs-mpAECs). Colors represent the volumetric depth of the sections at different distances from the front surface in voxel scale elaborated by the ReViSP.

The formation of three-dimensional structures was also tested by culturing cells in low-adherence round-bottom plates, and the cellular aggregation was followed by means of an automated live-cell imaging system for 7 days. After seeding in the low-attachment round-bottom plates, the cells began to self-assemble within 24 h, generating a spherical compact shape structure when mpMECs were in co-culture with vascular-wall mesenchymal stem cells (mpMECs–mpVW-MSCs) and in triple co-culture with vascular endothelial and mesenchymal stem cells (mpMECs–mpVW-MSCs–mpAECs) (Figure 3b–c’’’’). In monocultures of mpMECs, the formation of multiple smaller and irregularly shaped aggregates was also confirmed (Figure 3a–a’’’’). Brightfield object area analysis pointed out the significant differences between mpMECs monocultures and co-cultures and triple co-cultures (Figure 4a), as also demonstrated by the volumetric reconstruction (Figure 4c). No difference was evidenced at the two seeding densities, 5 × 10^3^ or 10 × 10^3^ cells/well. The eccentricity analysis, which measures how round and compact objects are in a range of 0 and 1, was determined by the Brightfield object average eccentricity parameter and revealed a more spherical structure in co-culture and triple co-culture; moreover, a significant difference was observed between the three groups analyzed (Figure 4b).

### 3.3. Spheroid Viability

**Figure 5:**
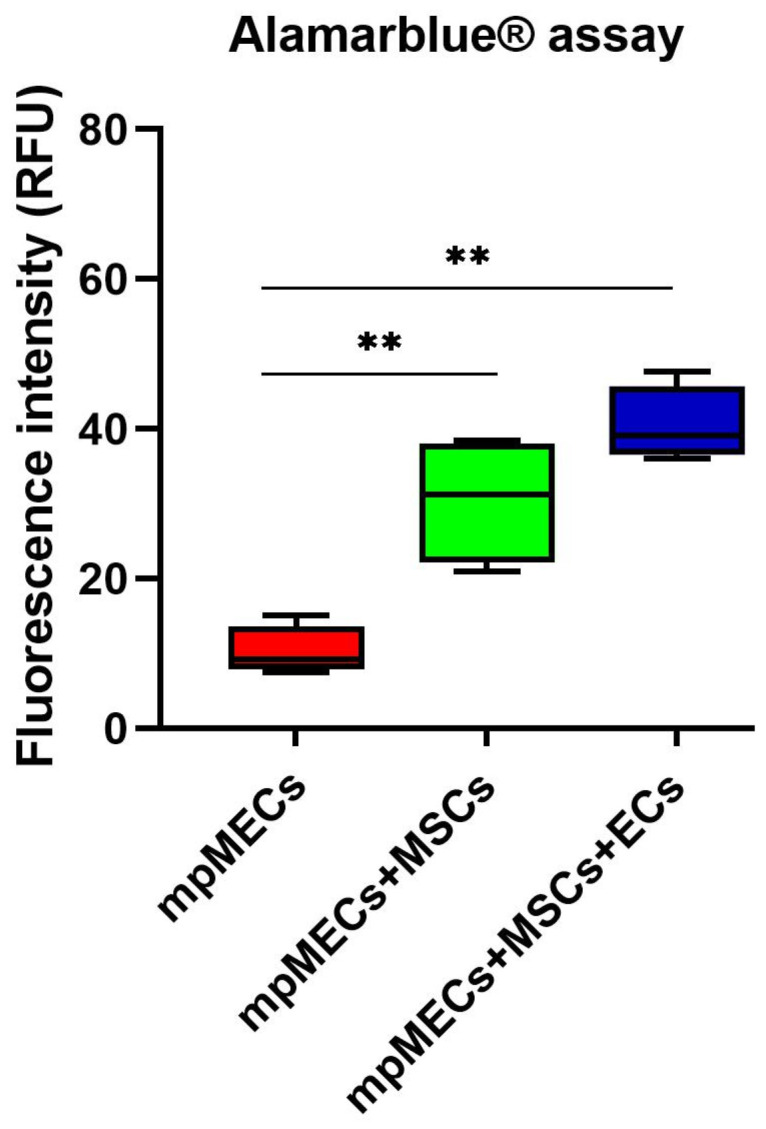
Viability assay of monoculture mpMECs and co-culture with the vascular-wall cell populations mpVW-MSCs and mpAECs after 8 days, measured with Alamarblue^®^ assay. The graph is represented as box and whiskers of the fluorescence intensity (RFU) of the resorufin measured on three independent experiments, and (**) *p* < 0.01, where the two segments that delimit the rectangle represent the 25th and 75th percentiles; the central segment is the median, and the bars are the minimum and maximum values, respectively.

The viability was evaluated after monitoring the spheroid formation in live-cell imaging at 8 days. Alamarblue^®^ assay viability test resulted in a higher metabolic activity of the cells in co- and triple co-cultures with respect to mpMECs monocultures (Figure 5).

### 3.4. Cellular Distribution of the mpVW-MSCs, mpAECs, and mpMECs on Multicellular Spheroid

**Figure 6:**
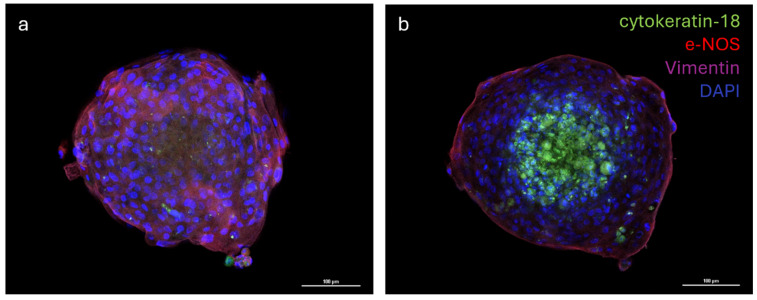
Immunostaining of multicellular spheroid of cytokeratin-18, endothelial nitric oxide synthase (e-NOS), and Vimentin; nuclei were counterstained with DAPI (**a**,**b**). Emission peaks were as follows: 470 nm for DAPI, 517 nm for cytokeratin-18 (FITC), 590 nm for e-NOS (Rhodamine Red X), and 665 nm for Vimentin (Alexa Fluor 647). Size: z 1/20 (**a**), 7/20 (**b**), step 1.2 µm; objective 20×.

The distribution of the three different cell populations was evaluated via the immunofluorescence analysis of multicellular spheroids. The analysis of images revealed that cells formed a well-organized three-dimensional structure where the vascular endothelial cells (e−NOS positive) and vascular-wall mesenchymal stem cells (vimentin positive) were distributed in the outermost part of the spheroid, while the mammary epithelial cells (cytokeratin−18 positive) were located in the inner part of the spheroid (Figure 6a,b).

## 4. Discussion

To faithfully reproduce a more complete in vitro model, resembling the mammary alveolus, the present work aims to develop a multicellular tri-dimensional (3D) model by co-culturing mammary epithelial cells (mpMECs) with two vascular-wall cell populations: the mesenchymal stem cells (mpVW-MSCs) and the aortic endothelial cells (mpAECs) derived from Göttingen minipigs. Cultures of mpVW-MSCs and mpAECs were previously simultaneously isolated from thoracic aorta tissues of the same Göttingen minipigs, to promote a more useful 3D in vitro heterotypic model [31]. Primary cultures of mpVW-MSCs showed a typical perivascular cell morphology, mpAECs a polygonal endothelial morphology, and mpMECs a cobblestone epithelial morphology. Different attempts have been conducted to recreate a mammary 3D model closer to the in vivo tissue using bovine and other livestock animals [25,26,27]. In the present study, two different methods were applied to find the better 3D cell culture system in terms of culture time, spheroids formation, and viability: the traditional hanging-drop cell culture method and the culture with ultra-low adherent low-bottom plates (ULA). When cultured with hanging-drop method, mpMECs showed the ability to aggregate, either alone or in triple culture, forming three-dimensional structures that are irregular in shape and of various sizes. The three cell populations aggregated similarly, forming 3D structures of different sizes within 24 h with a tendency to degenerate after 48 h. This is in contrast with what was reported by Ogorevc et al. [27] for primary goat mammary epithelial cells that formed spherical structures called ‘mammospheres’ after several days of culture by hanging drops. However, these differences could be attributed not only to the species-specific differences, but also to differences in the culture medium composition used. Also, a well-known bovine mammary epithelial cell line (BME-UV1) was reported to form polarized acinar structures, named mammospheres within 16 days, but when grown with Matrigel. These structures exhibited polarized epithelial cells in direct contact with basement membrane components using the tight junction proteins ZO-1 and epithelial-cadherin [34]. The effect of Matrigel and ultra-low-attachment (ULA) culture support vs. standard 2D cell culture was also compared on both the cell phenotypes and morphologies for the two most popular bovine mammary epithelial cell lines, MAC-T and BME-UV1, revealing differences [26]. MAC-T cells formed aberrant structures in ULA, while BME-UV1 cells showed a cluster of 3D sphere-like cells [26]. Few studies involving 3D culture of mammary cells have been conducted in the pig species, reporting the isolation of porcine MECs and the expression of milk protein gene transcripts, caseins, and ß-lactoglobulin, when cultivated in the presence of a matrix gel [25,35]. When cultured in low-adherence round-bottom plates, monocultures of mpMECs formed irregularly shaped aggregates, while VW-MSCs contributed to form a more compact spheroid within 24 h, also maintained in the triple culture with ECs. VW-MSCs have the peculiarity to structurally support the development of more compact spheroids; in fact, VW-MSCs interact physically with endothelial cells through contact junctions [15], and through the secretion of bioactive molecules, the vascular native microenvironment is recreated [15]. In addition, the spheroids containing VW-MSCs and endothelial cells resulted in higher viability after 8 days. Similar results have been reported in the co-culture of epithelial cells of amniotic origin with Wharton’s jelly mesenchymal stromal cells by Paris and colleagues [32]. Monocultures of epithelial cells do not self-assemble in spheroids but form a sheet of cells that easily dissociates. On the contrary, when co-cultured with a population of Wharton’s jelly mesenchymal stem cells, the cells self-assembled by forming compact and rigid spherically shaped spheroids with enhanced viability [32]. However, while the previous study has described co-culture models involving two cellular populations, the present work establishes a more complex and stable triple co-culture model comprising the mammary epithelial cells, the vascular wall-derived mesenchymal stem cells, and the endothelial cells, offering a more comprehensive in vitro approach to mimic the complexity of the mammary alveolar niche. Other heterotypic 3D co-culture models of luminal and myoepithelial cells, involving breast cancer cells and fibroblasts, stromal cells, or adipocytes, were investigated to explore the molecular-based mechanisms involved during human mammary gland development and carcinogenesis [36,37,38,39]. Similarly, to study the cellular spatial distribution and cancer invasion, heteromulticellular 3D spheroids consisting of epithelial cancer cell line models, microvascular endothelial cells, and stromal cells were combined in a scaffold-free culture system, showing that the onset of budding structures in co-cultured spheroids led to a significant decrease in spheroid circularity [40]. By closely mimicking the native tissue architecture and cellular microenvironment, the in vitro multicellular heterotypic 3D model proposed in the present research will enable precise investigation of lactation mechanisms, including hormonal regulation and milk protein synthesis. Additionally, the model could be useful as a platform for exploring the pathogenesis of mammary diseases, such as mastitis, facilitating the identification of early biomarkers and evaluation of therapeutic strategies [25]. Furthermore, by examining the cellular distribution within the spheroid, we observed the formation of a well-organized 3D structure. In this model, the vascular-wall-derived cells, specifically endothelial cells and mesenchymal stem cells, are distributed externally. Conversely, the mammary epithelial cells were situated internally, thereby mimicking the mammary alveolus structure where the secretory epithelium is enveloped by myoepithelial cells and blood capillaries [41]. Our results are consistent with a non-lactating cellular model, as the cells do not form the lumen (milk-filled) space. Further investigations are necessary to study the cellular hormonal modulation to further explore the lumen-polarized structures and changes in the gene expression profile. Since it was demonstrated that hormonal stimulation induces the complete maturation of epithelium, including the formation of cell junctions that allow for a correct cell polarization, the multicellular spheroid will be exposed to different concentrations of prolactin for different times to detect the formation of the functionally mature cavitated alveolus [22,25]. This will allow us to produce a more useful 3D in vitro model for a more complete blood–milk barrier study.

## 5. Conclusions

In conclusion, although the complete reconstitution of a fully functional mammary gland model in domestic species remains a challenge, our triple culture system could provide a novel and promising foundation for advancing in vitro studies of mammary tissue development in the porcine species. By integrating the mammary epithelial, vascular endothelial, and mesenchymal cellular components, this model better reflects the cellular complexity of the mammary niche and holds strong potential for applications in veterinary research, including lactation biology, mastitis studies, and regenerative approaches.

## Figures and Tables

**Table 1:** List of antibodies used for immunofluorescence analysis of spheroids.

Antibody	P. Number	Species	Supplier	Dilution
Primary				
eNOS	SA-258	mouse	BIOMOL Research Laboratories	1:100
FITC cytokeratin-18	ab52459	mouse	Abcam	5 µg/mL
Alexa-Fluor 647 vimentin	ab194719	mouse	Abcam	1:800
Secondary				
Rhodamine Red-X anti-mouse	AB_2340832	donkey	Jackson ImmunoResearch	1:800

Abbreviations: eNOS, endothelial nitric oxide synthase; FITC, fluorescein isothiocyanate.

## Data Availability

Data related to the area obtained by hanging drop, ultra-low attachment methods, eccentricity parameter, and viability are available on AMSActa Institutional Research Repository by AlmaDL University of Bologna Digital Library: Available online: https://amsacta.unibo.it/id/eprint/8506 (accessed on 21 October 2025).

## References

[1] Maldonado V.V., Patel N.H., Smith E.E., Barnes C.L., Gustafson M.P., Rao R.R., Samsonraj R.M. (2023). Clinical Utility of Mesenchymal Stem/Stromal Cells in Regenerative Medicine and Cellular Therapy. J. Biol. Eng..

[2] Margiana R., Markov A., Zekiy A.O., Hamza M.U., Al-Dabbagh K.A., Al-Zubaidi S.H., Hameed N.M., Ahmad I., Sivaraman R., Kzar H.H. (2022). Clinical Application of Mesenchymal Stem Cell in Regenerative Medicine: A Narrative Review. Stem Cell Res. Ther..

[3] Wu X., Jiang J., Gu Z., Zhang J., Chen Y., Liu X. (2020). Mesenchymal Stromal Cell Therapies: Immunomodulatory Properties and Clinical Progress. Stem Cell Res. Ther..

[4] Hoseinzadeh A., Esmaeili S.-A., Sahebi R., Melak A.M., Mahmoudi M., Hasannia M., Baharlou R. (2025). Fate and Long-Lasting Therapeutic Effects of Mesenchymal Stromal/Stem-like Cells: Mechanistic Insights. Stem Cell Res. Ther..

[5] Klein D. (2016). Vascular Wall-Resident Multipotent Stem Cells of Mesenchymal Nature within the Process of Vascular Remodeling: Cellular Basis, Clinical Relevance, and Implications for Stem Cell Therapy. Stem Cells Int..

[6] Dangat K., Khaire A., Joshi S. (2020). Cross Talk of Vascular Endothelial Growth Factor and Neurotrophins in Mammary Gland Development. Growth Factors.

[7] Bautch V.L. (2011). Stem Cells and the Vasculature. Nat. Med..

[8] Tang Z., Wang A., Yuan F., Yan Z., Liu B., Chu J.S., Helms J.A., Li S. (2012). Differentiation of Multipotent Vascular Stem Cells Contributes to Vascular Diseases. Nat. Commun..

[9] Psaltis P.J., Simari R.D. (2015). Vascular Wall Progenitor Cells in Health and Disease. Circ. Res..

[10] Invernici G., Emanueli C., Madeddu P., Cristini S., Gadau S., Benetti A., Ciusani E., Stassi G., Siragusa M., Nicosia R. (2007). Human Fetal Aorta Contains Vascular Progenitor Cells Capable of Inducing Vasculogenesis, Angiogenesis, and Myogenesis in Vitro and in a Murine Model of Peripheral Ischemia. Am. J. Pathol..

[11] Pasquinelli G., Tazzari P.L., Vaselli C., Foroni L., Buzzi M., Storci G., Alviano F., Ricci F., Bonafè M., Orrico C. (2007). Thoracic Aortas from Multiorgan Donors Are Suitable for Obtaining Resident Angiogenic Mesenchymal Stromal Cells. Stem Cells.

[12] da Silva Meirelles L., Chagastelles P.C., Nardi N.B. (2006). Mesenchymal Stem Cells Reside in Virtually All Post-Natal Organs and Tissues. J. Cell Sci..

[13] Howson K.M., Aplin A.C., Gelati M., Alessandri G., Parati E.A., Nicosia R.F. (2005). The Postnatal Rat Aorta Contains Pericyte Progenitor Cells That Form Spheroidal Colonies in Suspension Culture. Am. J. Physiol. Cell Physiol..

[14] Zaniboni A., Bernardini C., Alessandri M., Mangano C., Zannoni A., Bianchi F., Sarli G., Calzà L., Bacci M.L., Forni M. (2014). Cells Derived from Porcine Aorta Tunica Media Show Mesenchymal Stromal-like Cell Properties in in Vitro Culture. Am. J. Physiol. Cell Physiol..

[15] Zaniboni A., Bernardini C., Bertocchi M., Zannoni A., Bianchi F., Avallone G., Mangano C., Sarli G., Calzà L., Bacci M.L. (2015). In Vitro Differentiation of Porcine Aortic Vascular Precursor Cells to Endothelial and Vascular Smooth Muscle Cells. Am. J. Physiol. Cell Physiol..

[16] Luu N.T., McGettrick H.M., Buckley C.D., Newsome P.N., Rainger G.E., Frampton J., Nash G.B. (2013). Crosstalk between Mesenchymal Stem Cells and Endothelial Cells Leads to Downregulation of Cytokine-Induced Leukocyte Recruitment. Stem Cells.

[17] Vorwald C.E., Joshee S., Leach J.K. (2020). Spatial Localization of Endothelial Cells in Heterotypic Spheroids Influences Notch Signaling. J. Mol. Med..

[18] Meng X., Chen M., Su W., Tao X., Sun M., Zou X., Ying R., Wei W., Wang B. (2018). The Differentiation of Mesenchymal Stem Cells to Vascular Cells Regulated by the HMGB1/RAGE Axis: Its Application in Cell Therapy for Transplant Arteriosclerosis. Stem Cell Res. Ther..

[19] Bernardini C., Bertocchi M., Zannoni A., Salaroli R., Tubon I., Dothel G., Fernandez M., Bacci M.L., Calzà L., Forni M. (2019). Constitutive and LPS-Stimulated Secretome of Porcine Vascular Wall-Mesenchymal Stem Cells Exerts Effects on in Vitro Endothelial Angiogenesis. BMC Vet. Res..

[20] Lin R., Wang S., Zhao R.C. (2013). Exosomes from Human Adipose-Derived Mesenchymal Stem Cells Promote Migration through Wnt Signaling Pathway in a Breast Cancer Cell Model. Mol. Cell. Biochem..

[21] Klopp A.H., Lacerda L., Gupta A., Debeb B.G., Solley T., Li L., Spaeth E., Xu W., Zhang X., Lewis M.T. (2010). Mesenchymal Stem Cells Promote Mammosphere Formation and Decrease E-Cadherin in Normal and Malignant Breast Cells. PLoS ONE.

[22] McManaman J.L., Neville M.C. (2003). Mammary Physiology and Milk Secretion. Adv. Drug Deliv. Rev..

[23] Kobayashi K. (2023). Culture Models to Investigate Mechanisms of Milk Production and Blood-Milk Barrier in Mammary Epithelial Cells: A Review and a Protocol. J. Mammary Gland. Biol. Neoplasia.

[24] Kobayashi K., Oyama S., Numata A., Rahman M.M., Kumura H. (2013). Lipopolysaccharide Disrupts the Milk-Blood Barrier by Modulating Claudins in Mammary Alveolar Tight Junctions. PLoS ONE.

[25] Finot L., Chanat E., Dessauge F. (2021). Mammary Gland 3D Cell Culture Systems in Farm Animals. Vet. Res..

[26] Arévalo Turrubiarte M., Perruchot M.-H., Finot L., Mayeur F., Dessauge F. (2016). Phenotypic and Functional Characterization of Two Bovine Mammary Epithelial Cell Lines in 2D and 3D Models. Am. J. Physiol. Cell Physiol..

[27] Ogorevc J., Zorc M., Dovč P., Kukovics S. (2018). Development of an In Vitro Goat Mammary Gland Model: Establishment, Characterization, and Applications of Primary Goat Mammary Cell Cultures. Goat Science.

[28] Bernardini C., La Mantia D., Salaroli R., Zannoni A., Nauwelaerts N., Deferm N., Ventrella D., Bacci M.L., Sarli G., Bouisset M. (2021). Development of a Pig Mammary Epithelial Cell Culture Model as a Non-Clinical Tool for Studying Epithelial Barrier—A Contribution from the IMI-ConcePTION Project. Animal.

[29] Bernardini C., Nesci S., La Mantia D., Salaroli R., Nauwelaerts N., Ventrella D., Elmi A., Trombetti F., Zannoni A., Forni M. (2024). Isolation and Characterization of Mammary Epithelial Cells Derived from Göttingen Minipigs: A Comparative Study versus Hybrid Pig Cells from the IMI-ConcePTION Project. Res. Vet. Sci..

[30] La Mantia D., Nauwelaerts N., Bernardini C., Zannoni A., Salaroli R., Lin Q., Huys I., Annaert P., Forni M. (2024). Development and Characterization of a Human Mammary Epithelial Cell Culture Model for the Blood–Milk Barrier—A Contribution from the ConcePTION Project. Int. J. Mol. Sci..

[31] Bernardini C., La Mantia D., Salaroli R., Ventrella D., Elmi A., Zannoni A., Forni M. (2023). Isolation of Vascular Wall Mesenchymal Stem Cells from the Thoracic Aorta of Adult Göttingen Minipigs: A New Protocol for the Simultaneous Endothelial Cell Collection. Animals.

[32] Paris F., Marrazzo P., Pizzuti V., Marchionni C., Rossi M., Michelotti M., Petrovic B., Ciani E., Simonazzi G., Pession A. (2023). Characterization of Perinatal Stem Cell Spheroids for the Development of Cell Therapy Strategy. Bioengineering.

[33] Piccinini F., Tesei A., Arienti C., Bevilacqua A. (2015). Cancer Multicellular Spheroids: Volume Assessment from a Single 2D Projection. Comput. Methods Programs Biomed..

[34] Kozlowski M., Gajewska M., Majewska A., Jank M., Motyl T. (2009). Differences in Growth and Transcriptomic Profile of Bovine Mammary Epithelial Monolayer and Three-Dimensional Cell Cultures. J. Physiol. Pharmacol..

[35] Sun Y.L., Lin C.S., Chou Y.C. (2006). Establishment and Characterization of a Spontaneously Immortalized Porcine Mammary Epithelial Cell Line. Cell Biol. Int..

[36] Darcy K.M., Zangani D., Lee P.-P.H., Ip M.M., Ip M.M., Asch B.B. (2000). Isolation and Culture of Normal Rat Mammary Epithelial Cells. Methods in Mammary Gland Biology and Breast Cancer Research.

[37] Shekhar M.P., Werdell J., Tait L. (2000). Interaction with Endothelial Cells Is a Prerequisite for Branching Ductal-Alveolar Morphogenesis and Hyperplasia of Preneoplastic Human Breast Epithelial Cells: Regulation by Estrogen. Cancer Res..

[38] Proia D.A., Kuperwasser C. (2006). Reconstruction of Human Mammary Tissues in a Mouse Model. Nat. Protoc..

[39] Wang X., Kaplan D.L. (2012). Hormone-Responsive 3D Multicellular Culture Model of Human Breast Tissue. Biomaterials.

[40] Ortiz E., Thway K.H., Ortiz-Soto G., Yao P., Kelber J.A. (2025). Heteromulticellular Stromal Cells in Scaffold-Free 3D Cultures of Epithelial Cancer Cells to Drive Invasion. J. Vis. Exp..

[41] Masedunskas A., Weigert R., Mather I. (2014). Intravital Imaging of the Lactating Mammary Gland in Transgenic Mice Expressing Fluorescent Proteins.

